# Development and validation of a home literacy questionnaire for Malayalam-speaking preschoolers

**DOI:** 10.3389/fpsyg.2026.1841280

**Published:** 2026-06-15

**Authors:** Vellithiruthi Thazhath Aaruni, Radhakrishnan Chella Perumal, Murugesan Krupa, Jayashree Chandrashekhar Shanbal, Arjunan Vasanthy Anjana

**Affiliations:** 1 Sri Ramachandra Institute of Higher Education and Research, Chennai, India; 2 All India Institute of Speech and Hearing, Mysuru, India; 3 National Institute of Speech and Hearing, Thiruvananthapuram, India

**Keywords:** good health and wellbeing, home literacy environment, home literacy questionnaire, Malayalam, quality education

## Abstract

**Introduction:**

Environmental contexts, particularly the home literacy environment (HLE), play a significant role in shaping children’s early literacy development. Despite this, there are no validated tools available in Malayalam, the primary language spoken in Kerala, India, for assessing key environmental factors. Therefore, this study aimed to develop and validate the Home Literacy Questionnaire (HLQ) for Malayalam speaking preschool children.

**Method:**

Questionnaire items were developed based on established frameworks of the HLE and underwent content validation by the experts. The content validity of the questionnaire was evaluated using the content validity index (CVI), including both item-level (I-CVI) and scale-level (S-CVI) indices. Based on the CVI results and expert feedback, necessary revisions were made, and it was subsequently administered to 300 parents. Percentage analysis was employed to examine the distribution of responses for each questionnaire item. Confirmatory factor analysis (CFA) was performed to evaluate the construct validity. Internal consistency was assessed using Cronbach’s alpha.

**Results:**

The HLQ demonstrated satisfactory content validity, with acceptable I-CVI and S-CVI values. CFA results indicated an acceptable model fit, supporting the construct validity of the questionnaire. The scale also showed good internal consistency, with satisfactory Cronbach’s alpha values. Response patterns provide insights into parental reading practices, child literacy habits, parental literacy beliefs, parent child literacy interactions and the physical environment within the home.

**Discussion:**

The findings suggest that that the HLQ is a valid and reliable instrument for assessing key dimensions of the home literacy environment among Malayalam speaking preschool children. The questionnaire offers a comprehensive means of evaluating literacy related practices and resources within the home and may facilitate future research examining the influence of the HLE on children’s literacy development.

## Background

“Early literacy” encompasses the development of foundational knowledge, skills, and attitudes related to reading, writing, and language that emerges before formal literacy instruction (Justice and Ezell, 2004). The development of early literacy skills is mediated by children’s experiences and environments in both home (Zucker and Grant, 2007) and school settings (Ezell and Justice, 2005). The home literacy environment (HLE) is a broad construct encompassing the language and literacy resources, interactions, and practices that children are exposed to within the home setting (Hamilton et al., 2016). A growing body of research examining the HLE as a predictor of child development has highlighted that the quality of the HLE significantly supports children’s cognitive and socio-emotional development (Kluczniok and Mudiappa, 2019). Examining the influence of the literacy environment is crucial to understanding the contextual factors that affect children’s literacy development. However, the absence of standardized tools in Malayalam constrains empirical investigation in this area.

The HLE is a diverse and intricate concept (Burgess et al., 2002; Weigel et al., 2006), typically characterized by two main components: the availability of literacy resources and the nature of literacy-related activities within the home (Grolig, 2020). The first component primarily pertains to the availability and accessibility of reading materials for both children and their parents (Li and Li, 2022; Zhang et al., 2020), which plays a crucial role in fostering the development of children’s oral language and code-related abilities (Burris et al., 2019; De Bondt et al., 2020). The second encompasses both active and passive aspects of the HLE (Burgess et al., 2002). Active HLE involves children’s direct participation in literacy-oriented activities, whereas passive HLE reflects children’s incidental learning through observing parents’ literacy behaviors, such as reading or writing practices. Van Steensel (2006) identified three distinct types of HLE: (a) rich HLE, characterized by frequent and diverse literacy-related interactions between parents and children, (b) child-focused HLE -where the range of literacy activities is relatively limited but includes meaningful experiences such as shared reading and library visits, and (c) poor HLE - marked by minimal engagement of both the parents and children in literacy activities.

Previous research has widely documented five key dimensions of the HLE: (a) physical environment, referring to the availability of books and print materials within the home (Curenton and Justice, 2008; Niklas, 2015), (b) parent literacy practices encompassing parents’ own reading and writing habits (Nunley, 2000), (c) child’s own literacy habits reflecting their self-initiated engagement with print (Deckner et al., 2006; Justice et al., 2002), (d) parent-child interactions for literacy-related activities involving shared reading, storytelling, and other literacy based exchanges (Masudi and Silaji, 2024), (e) parental beliefs and attitudes toward literacy (Bergman Deitcher et al., 2024; Wu and Hindman, 2025).

The literature reports that home literacy activities differ in high-income and low-income nations. In a study of low- and middle-class Koreans, parents reported they read books to their children once a week, assisted them with their schoolwork three to four times a week, and taught them literacy and the Korean alphabet letters and symbols. However, they only took their children to the library or bookstore once a month (Kim, 2009). According to a study conducted in Japan, parents read to their first-graders for an average of five to thirty minutes each day and taught them character and kanji names, word reading, and character writing a few times a month (Inoue et al., 2018). As evidenced by studies from Indonesia (Mayasya, 2017) and Cambodia (Howell et al., 2016), families in some regions value direct literacy instruction over storybook reading and storytelling.

Research from Asian nations reveals that mothers are more likely to report engaging in home learning activities with their children than fathers (Sun et al., 2018). Sad and Gürbüztürk (2013) found that mothers in Turkey reported helping their children with homework more often than fathers did.

Lau and McBride-Chang (2005) observed that asking questions about the story’s content during parent-child reading was a strong predictor of second graders’ abilities to recognize Chinese characters in a study on shared reading activities in Hong Kong. Huang et al. (2021) reported during math-related application activities in China, preschoolers’ initiative-taking behaviors, such as coming up with answers on their own and persevering when challenges arise, were positively connected with mothers’ emotional support. Similarly, research indicates that parents’ attitudes and beliefs on education can affect children’s literacy and numeracy outcomes both directly and indirectly. Children’s interest in numeracy activities was positively correlated with parents’ attitudes toward numeracy, including their views about the ability to educate and the role they play in their education (Cheung et al., 2018).

Parent literacy practices and children’s own interest in engaging in literacy-related activities can also influence children’s reading outcomes. In India, children’s vocabulary scores at the end of the year were correlated with adult literacy practices (Vagh, 2009). Children in Bangladesh were more likely to do well on reading assignments if they saw more than three family members reading at home (Islam et al., 2018). Parents’ literacy levels were strongly correlated with their children’s literacy in Nepal and the Philippines (LeVine et al., 2012).

There is a paucity of studies examining the influence of environmental factors on early literacy skills in India, particularly in Kerala. This gap is largely attributable to the limited availability of assessment tools that are linguistically and culturally appropriate for Malayalam-speaking children. Although a home literacy questionnaire was previously developed in Tamil (Buvaneswari and Padakannaya, 2017), it did not account for crucial aspects such as the number of books available at home, the age at which books are introduced, or the duration and frequency of shared reading, factors essential for understanding the depth and consistency of children’s literacy experiences. The present study, therefore, aimed to address these limitations by developing a questionnaire that is culturally and linguistically relevant to Malayalam-speaking populations. Malayalam, the official and most widely spoken language of Kerala in southern India, is rooted in a region distinguished by a literacy rate of 96.2%, one of the highest in the country. Using a literacy questionnaire in Malayalam allows respondents to engage with the content in a more familiar and meaningful way, promoting clear understanding, more authentic responses, and greater ease in participation. In turn, the present study developed and validated home literacy questionnaire to assess the literacy environments of Malayalam-speaking preschoolers across the home context.

## Materials and methods

This study is part of ongoing doctoral research examining the development of early literacy skills and the influence of environmental factors among 300 Malayalam-speaking preschool children. A cross-sectional study design was used. The participants in the present study were 300 parents of Malayalam-speaking children aged 3–6 years. Institutional Ethics Committee approval was obtained before the commencement of the study (Ref: IEC-NI/23/MAY/87/28). Participants were informed about the purpose of the study, and written informed consent was obtained before proceeding with the study.

### Development and validation of the home literacy questionnaire (HLQ)

To evaluate the literacy environment in home settings, a questionnaire was developed in Malayalam. The development of questionnaire followed a systematic process that included generating items, refining their wording and organizing them in a coherent sequence. Items for HLQ were derived following an extensive review of the literature on the HLE (Buvaneswari and Padakannaya, 2017; Foy and Mann, 2003; Martini, 2004; Pandith et al., 2022). The development of the questionnaire was grounded in established theoretical frameworks of the HLE. The selection of items was guided by five core aspects of the home literacy context: physical environment, parent literacy habits, child literacy habits, parent-child interaction for literacy-related habits, parental beliefs and perceived barriers toward early literacy, as well as based on the age and regularity of reading, comprising 44 items. Out of these, 6 items pertained to the age and regularity of reading, 6 items belonged to the physical environment at home, 8 items each represented parent literacy habits, child literacy habits, parent-child interaction for literacy-related activities, and parental beliefs about literacy.

The physical environment refers to the availability of literacy resources and spaces within the home. This dimension included six items assessing the presence of different types of books in the household, such as board books, puzzle books, and concept books. The items also assessed whether these books are easily accessible to children and whether a designated storage place is available in the home.

Parent literacy habits refer to personal interests and reading habits of parents. It included items that assessed parents, personal interest in reading books, magazines or newspapers; their use of expressive voice and finger tracking while reading; whether they like discussing books they read with others at home; their interest in rereading books, whether they enjoy reading aloud to others; and whether children observe them engaging in reading-related activities.

Child literacy habits are the interest and participation of children in reading-related activities. This dimension includes items assessing children’s interests in books and reading; requests to read aloud, and their active engagement during shared reading experiences. It also examined whether a child asks questions when the story is unclear and follows along when an adult is reading a book.

Parent-child interaction refers to the literacy-related interactions and shared activities between adults and children. This dimension included items assessing whether parents relate stories to real life to make easily understandable; create new stories or songs for children; explain the content while watching television together; and whether parents encourage children to read environmental prints.

Parental beliefs and perceived barriers toward literacy refers to parent’s attitudes, perceptions, and views regarding their role in supporting children’s literacy development as well as the challenges that limit their involvement in literacy related activities. This dimension included items assessing their belief about importance of early reading, perceptions of their child’s readiness and interest in reading and barriers such as lack of time, absence of peaceful reading environment at home, and difficulties in maintaining children’s attention during reading activities.

The questionnaire included 38 closed-ended items with binary yes/no response options and 6 multiple-choice questions with four options. Multiple-choice items were used only to gather specific information related to age and regularity of reading. These items addressed the factors such as the age at which parents introduced books to their children, the number of books available in their households, and the duration and frequency of shared book-reading activities. The questionnaire also elicited gender related information related to the father’s and mother’s education qualifications, occupation, and socioeconomic status. However, age-related information of the respondents was not sought out in the literacy questionnaire.

The developed HLQ underwent content validation. The panel of raters comprised three speech language pathologists, each possessing a minimum of 5 years of clinical experience in the assessment and management of childhood language and literacy disorders. Although the content validation was performed by a relatively small expert panel, all three experts possessed the relevant clinical and research expertise necessary for validating the questionnaire. Comprehensive instructions were provided to the raters on evaluating the relevance of each item within its respective domain. They rated the items using a four-point Likert scale ranging from 1 (not relevant) to 4 (highly relevant). A separate section was included for raters to record their remarks, suggestions, or observations related to particular items or dimensions. Prior to rating, a detailed orientation session was held to ensure a clear and consistent understanding of the validation procedure among all experts.

### Administration of the questionnaire

Questionnaires were primarily distributed through the respective class teachers; however, when parents were available, they were approached directly and requested to complete the form in person. Parents of children aged 3–4 years from 17 Anganwadi centers, and parents of children aged 4–6 years from three schools managed by the Kerala state government participated as respondents. An Anganwadi is a community-based child care center in rural India, established under the Integrated Child Development Services (ICDS) program to address child hunger, malnutrition, and early developmental needs. Although the home literacy experiences begin earlier in childhood, the present study focused on children aged 3–6 years, as it was conducted as part of ongoing doctoral research examining the development of early literacy skills among preschool children within this age range. One parent of each child completed the questionnaire; however, the specific respondent (mother/father) was not separately documented during data collection. All participants were provided with clear instructions to read each item carefully and record their responses. Informed consent was obtained before administration, and participants were encouraged to seek clarification for any queries related to the questionnaire. Most of the participants completed the questionnaires within 2 days.

#### Scoring and analysis

Statistical analysis was conducted using Jamovi. Percentage analysis was employed to examine the distribution of responses for each questionnaire item. All “yes” responses were scored “1” and all “no” responses were scored “0.” Negatively worded items in the parental beliefs and perceived barriers domain were reverse-coded before analysis. Content validity was established using the content validity index, including both item-level (I-CVI) and scale-level (S-CVI) indices. Construct validity was examined using the confirmatory factor analysis (CFA) to test the hypothesized five-factor structure derived from the theoretical framework of the HLE. Model fit was evaluated using multiple indices, including the chi-square, comparative fit indices (CFI), Tucker-Lewis Index (TLI), root mean square error of approximation (RMSEA), and standardized root mean square residual (SRMR). Internal consistency reliability was assessed using Cronbach’s alpha coefficients.

Education, occupation, and socioeconomic details of participants are presented in Table 1.

**TABLE 1:** Education, occupation and socioeconomic details of participants (*N* = 300).

Education, occupation and socioeconomic status	Father (n)	Father (%)	Mother (n)	Mother (%)
Educational qualification				
Up to middle school High school Higher secondary Degree/Diploma PG or above	11 189 62 33 5	3.66 63 20.66 11 1.66	1 70 119 92 18	0.33 23.33 39.66 30.66 6
Occupation				
Unemployed Elementary occupation Skilled workers Professionals Senior officials	1 124 160 12 3	0.33 41.33 53.33 4 1	264 4 14 18 0	88 1.33 4.66 6 0
Socioeconomic status				
Lower middle Upper lower Upper middle Upper	86 132 74 8		28.66 44 24.66 2.66	

## Results

### Content validation of the questionnaire

The data collected from the experts were subjected to statistical analyses to estimate the content validity. The I-CVI values for the HLQ items ranged from 0.6 to 1. Items that obtained an I-CVI of 0.6 were subsequently revised to enhance clarity and relevance, whereas items with higher agreement were retained. The S-CVI/Ave values across the HLQ domains ranged from 0.80 to 1.00, indicating acceptable to excellent scale-level content validity. A kappa value of 0.724 demonstrates good inter-rater agreement among the experts. Detailed item-wise content validity indices are provided in Supplementary Table 1.

#### Construct validity

CFA was conducted to examine the five-factor structure of the HLQ in a sample of 300 parents. The hypothesized model consisted of five latent constructs: physical environment, parent literacy habits, child literacy habits, parent-child interaction for literacy-related activities and parental beliefs & perceived barriers for early literacy. The initial model evaluation identified four items from the parental beliefs construct (PB1, PB2, PB6, PB8), four items from parent-child interaction (PCI1, PCI2, PCI3, PCI4), three items from the child literacy habits (CLH5, CLH6, CLH7), and one item from parent literacy habits (PLH7) as weak indicators due to non-significant and low standardized factor loadings (<0.30). Although these were theoretically relevant, they were removed due to weak psychometric performance. Future research may refine and re-evaluate these items. Modification indices were examined, and error covariances were added between theoretically related items within the same constructs (PE4∼PE6), as these items are closely related literacy behaviors.

The final model demonstrated acceptable fit to data χ^2^(288) = 396, *p* < 0.001, comparative fit index (CFI) = 0.903, Tucker Lewis Index (TLI) = 0.890, Standardized Root Mean Square Residual (SRMR) = 0.0524, and Root Mean Square Error of Approximation (RMSEA) = 0.0353. All retained items loaded significantly onto their respective latent constructs (*p* < 0.001). Standardized factor loadings ranged from 0.319 to 0.818 as represented in Table 2. Physical environment items showed loadings from 0.319 to 0.515, parent literacy habits from 0.330 to 0.637, child literacy habits from 0.330 to 0.567, parent-child interaction from 0.387 to 0.631 and parental beliefs from 0.497 to 0.818.

**TABLE 2:** Standardized factor loadings of the HLQ factors.

Factor	Item	Standardized loading
Physical environment (PE)	PE1 PE2 PE3 PE4 PE5 PE6	0.417 0.319 0.515 0.427 0.510 0.339
Parent literacy habits (PLH)	PLH1 PLH2 PLH3 PLH4 PLH5 PLH6 PLH8	0.367 0.626 0.343 0.637 0.548 0.483 0.330
Child literacy habits (CLH)	CLH1 CLH2 CLH3 CLH4 CLH8	0.501 0.531 0.461 0.567 0.330
Parent-child interaction for literacy-related activities (PCI)	PCI5 PCI6 PCI7 PCI8	0.631 0.434 0.413 0.387
Parental beliefs and perceived barriers toward early literacy (PB)	PB3 PB4 PB5 PB7	0.704 0.818 0.622 0.497

*p* < 0.001.

**FIGURE 1:**
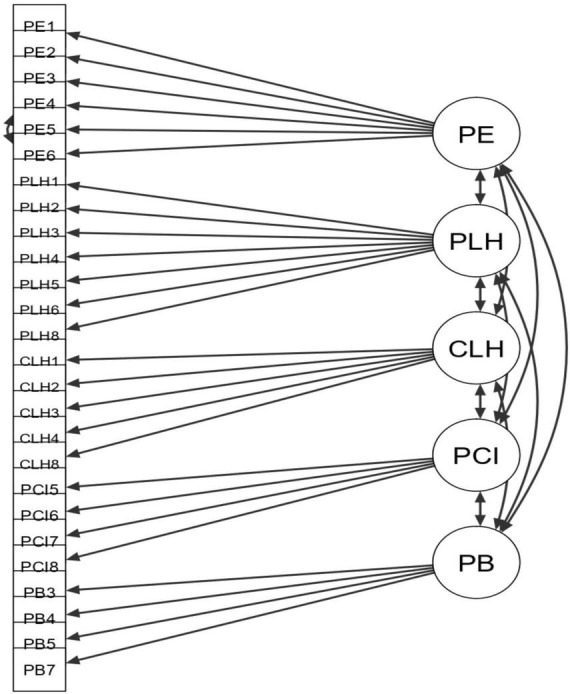
Confirmatory factor analysis model of HLQ. PE, Physical environment; PLH, Parent literacy habits; CLH, Child literacy habits; PCI, Parent-child interactions for literacy-related activities; PB, Parental beliefs & perceived barriers toward early literacy.

The graphical representation of the model is shown in Figure 1. It illustrates the relationships between the five latent constructs and their respective observed indicators.

Cronbach’s alpha was used to assess the internal consistency of the HLQ; values ranged from 0.524 to 0.756. The coefficients for the individual domains were 0.53 for PE, 0.671 for PLH, 0.591 for CLH, 0.524 for PCI and 0.756 for PB. Relatively low Cronbach’s alpha value can occur in newly developed and multi-dimensional tool, especially when it assesses multiple constructs rather than single domain (Cho and Kim, 2015; Panayides, 2013).

### Responses of the participants

#### Age and regularity of reading

As indicated in Table 3, nearly half of the respondents, 143 (47.66%), reported commencing book exposure at or after the age of 3. For the recent book activity, 140 (46.66%) had read to their children the previous day. Of the participating parents, 171 (57%) engaged in reading with their children at least one to three times a week. Parents reported considerable variation in the time they spent reading with their children. The most common duration, noted by 127 parents (42.33%), was approximately 10 min per session. Picture story books were the most preferred reading material, as indicated by 112 parents (37.33%). About the number of books available at home, 217 parents (72.33%) reported having fewer than ten books.

**TABLE 3:** Frequency distribution of age and regularity of reading.

Questions related to age and regularity of reading	Choices	*n* (%)
Age at which you started reading books to your child	Less than 1 year of age	5 (1.66)
	1–2 years	31 (10.33)
	2–3 years	121 (40.33)
	3 years and above	143 (47.66)
Last time you read books to your child?	Yesterday	140 (46.66)
	A few days before	87 (29)
	A few weeks before	37 (12.33)
	A few months before	36 (12)
How often are books read to your child in a week	1–3 times/week	171 (57)
	3–5 times/week	71 (23.66)
	5–7 times/week	41 (13.66)
	Never	17 (5.66)
How long have books been read to your child	Less than 5 min	51 (17)
	10 min	127 (42.33)
	Half an h	108 (36)
	More than 1 h	14 (4.66)
Preferred types of books for reading to your child	Short story books	61 (20.33)
	Picture story books	112 (37.33)
	Folk stories/moral stories	37 (12.33)
	Children’s magazines like Balarama, Balabhumi or Kalikkudukka	90 (30)
Number of children’s books available at home	None	20 (6.66)
	Less than 10 books	217 (72.33)
	Less than 20 books	42 (14)
	More than 20 books	21 (7)

#### Physical environment

**FIGURE 2:**
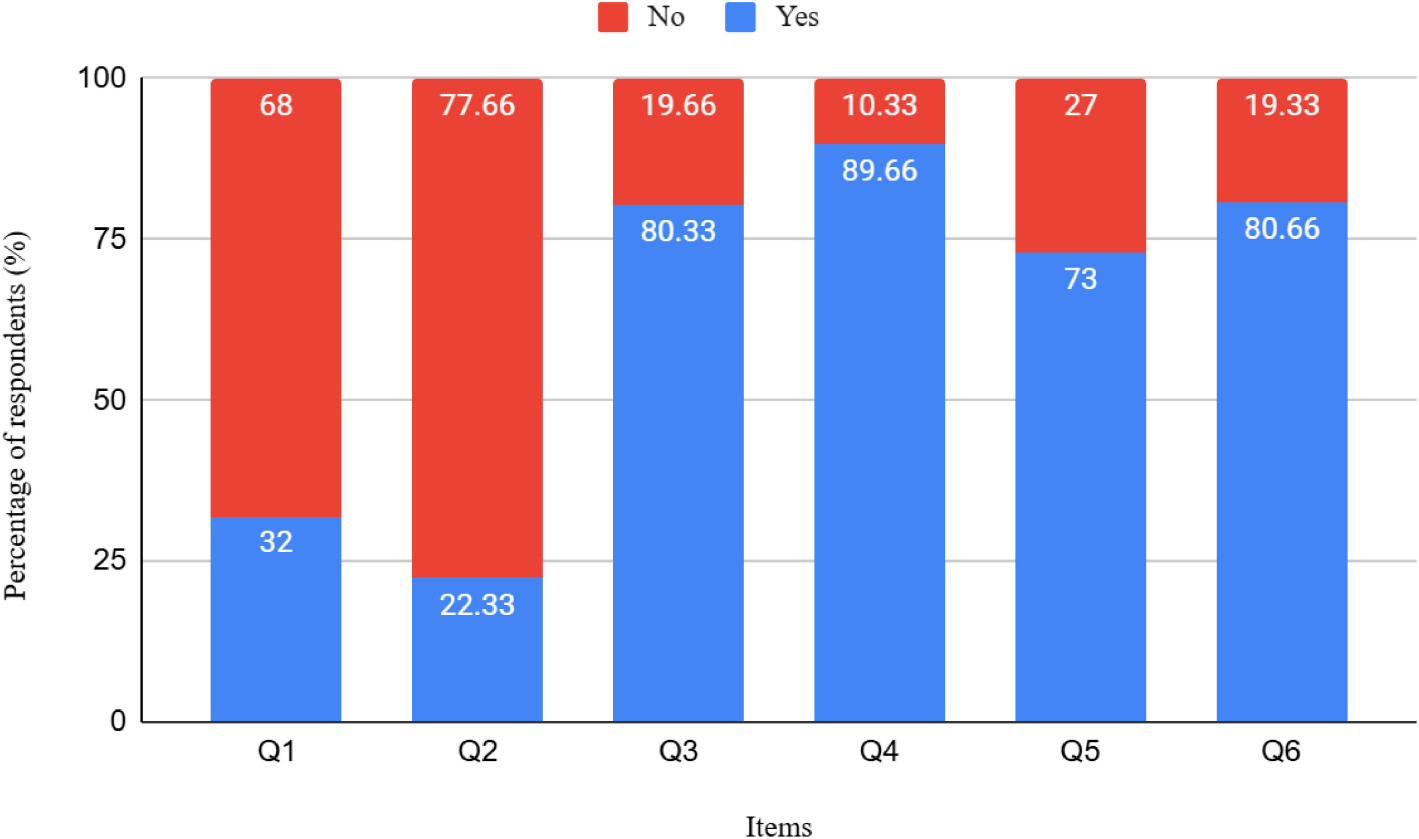
Parents’ responses to the physical environment domain. Q1, availability of board books; Q2, availability of puzzle books; Q3, concept books at home, Q4, alphabet and number books; Q5, accessibility of books; Q6, designated book space at home.

As represented in Figure 2, out of all participants, 96 (32%) reported using board books at home to teach early concepts such as shapes and colors (Q1). Additionally, 67 participants (22.33%) noted the availability of puzzle books in their households (Q2). A large majority of 241 parents (80.33%) reported availability and use of books that introduce concepts like animal names, body parts, fruit and vegetable names (Q3), while 269 (89.66%) stated that they have books to support learning numbers and alphabet (Q4). Furthermore, 219 (73%) noted that books are easily accessible to children at home (Q5), and 242 (80.66%) mentioned they have a designated space for storing books (Q6).

#### Parent literacy habits

**FIGURE 3:**
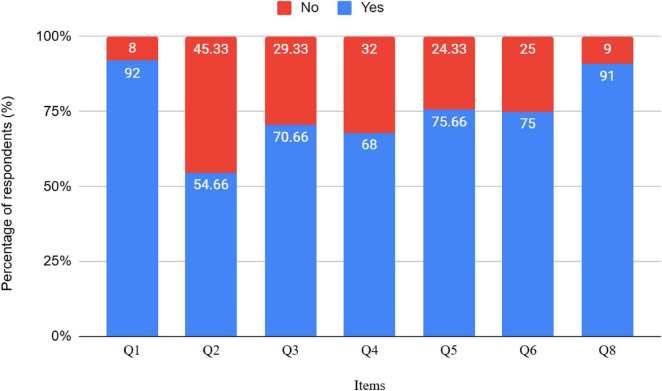
Parents’ responses to the parent literacy habits domain. Q1, interest in reading; Q2, voice variation while reading; Q3, finger tracking while reading; Q4, discussing books at home; Q5, re-reading books; Q6, enjoys reading aloud; Q8, child observes parental reading.

As shown in Figure 3, a significant proportion of parents, 276 (92%), expressed a personal interest in reading various materials such as books, newspapers, and magazines (Q1). Approximately 164 parents (54.66%) reported that they use expressive intonation or vary their voice when reading books (Q2). Moreover, 212 parents (70.66%) noted that they trace words with their fingers or point to them while reading (Q3). A total of 204 parents (68%) reported enjoying discussing books or stories they read with other family members at home (Q4). In addition, 227 parents (75.66%) stated that they like revisiting and rereading books they have previously enjoyed (Q5). 225 parents (75%) indicated they enjoy reading books aloud to others (Q6). And nearly 273 (91%) shared that their children observe them reading books or newspapers (Q8).

#### Child literacy habits

**FIGURE 4:**
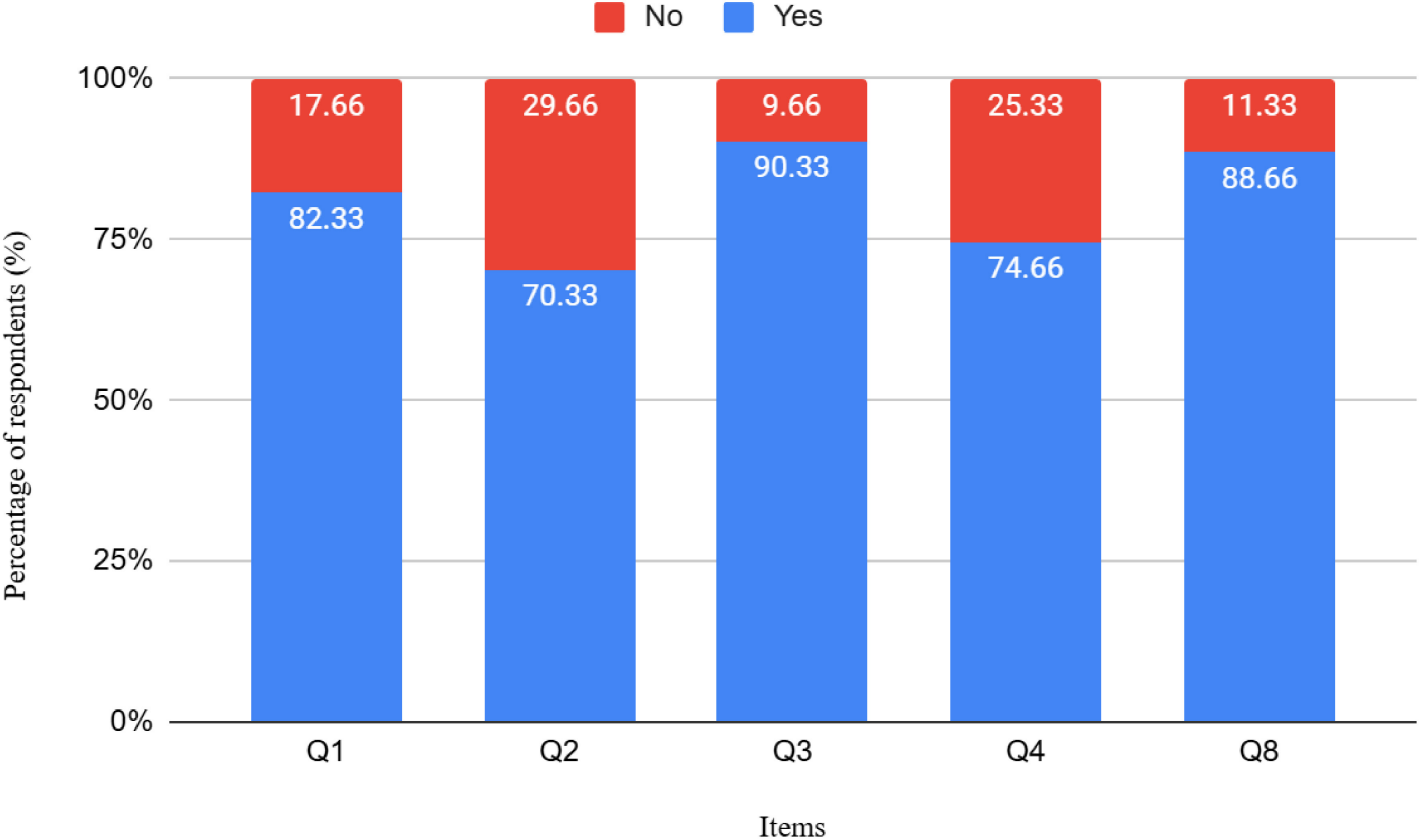
Parents’ responses to the child literacy habits domain. Q1, child’s interest in reading; Q2, requests for reading aloud; Q3, active reading engagement; Q4, asks questions during reading; Q8, follows along during reading.

The data represented in Figure 4 indicate that a majority of parents reported positive reading-related behaviors in their children. Specifically, 247 (82.33%) respondents indicated that their children show interest in reading (Q1), while 211 (70.33%) participants mentioned that their children often request them to read aloud (Q2). Furthermore, 271 (90.33%) observed that their children actively engage with books or magazines (Q3). Many parents also noted their children’s curiosity and initiative toward reading - 224 (74.66%) informants stated their children ask questions when they do not understand a story (Q4). Additionally, 266 (88.66%) responded that their children can effectively follow along during reading sessions (Q8). This underscores children’s strong interest, active engagement, and comprehension during reading activities, all of which are reinforced by meaningful and consistent parental involvement.

#### Parent-child interaction for literacy-related habits

**FIGURE 5:**
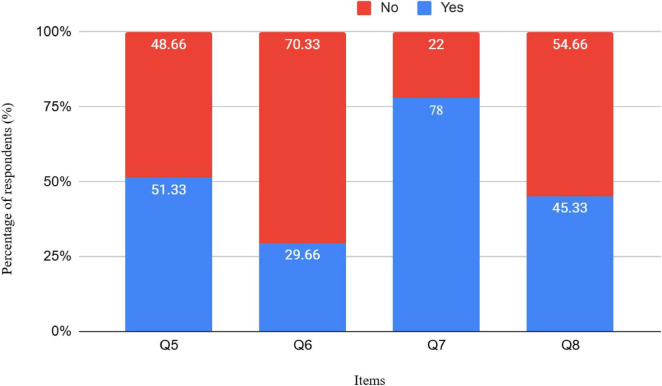
Parents’ responses to the parent-child interaction for the literacy-related activities domain. Q5, relating stories to real life; Q6, creating new stories/songs; Q7, discussing tv contents; Q8, encouraging environmental reading.

Figure 5 represents parent-child interaction for literacy-related activities. Around 154 parents (51.33%) shared that they relate stories to real-life situations to support better understanding (Q5). A comparatively lower number of parents, 89 (29.66%), create new stories or songs for their children (Q6). Additionally, 234 parents (78%) stated that they explain things to their children while watching television together (Q7), and 136 parents (45.33%) encourage their children to read signboards or product labels when they come across them (Q8).

#### Parental beliefs and perceived barriers toward early literacy

**FIGURE 6:**
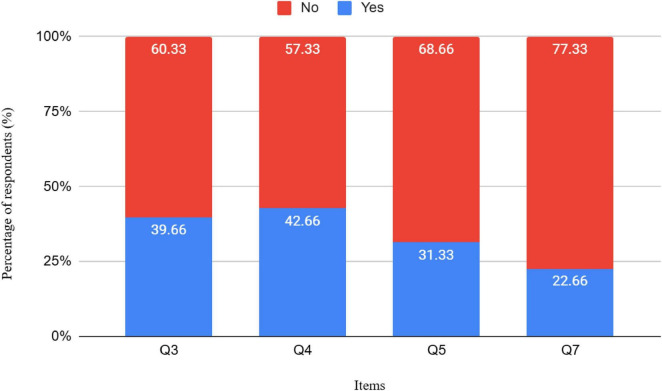
Parents, responses to the parental beliefs about the literacy domain. Q3, child doesn’t sit for reading; Q4, no peaceful reading environment at home; Q5, time constraints for reading; Q7, belief that reading should not be introduced to young children.

Parents expressed strong beliefs about their role in supporting early literacy and their perceived barriers toward reading, as indicated in Figure 6. Specifically, 119 (39.66%) indicated that they do not read to their children because the children do not sit for reading (Q3), 128 (42.66%) believe that the lack of a peaceful environment prevents them from reading with their children (Q4), and 94 (31.33%) believed that limited time makes shared reading difficult (Q5). Despite this, parents also strongly valued the benefits of reading; 232 (77.33%) held the opinion that children should begin reading at a young age (Q7).

## Discussion

This study investigated the five-factor structure of the HLQ in parents of preschool children. Empirically, the results provide clear psychometric evidence for a multidimensional view of the HLE, which includes the physical environment, parent literacy habits, child literacy habits, parent-child interaction for literacy-related activities and parental beliefs & perceived barriers for literacy. The final model demonstrated an acceptable fit to the data, indicating that the proposed factor structure represents the relationships among the observed variables. Several items were removed, suggesting that certain literacy behaviors and statements may not have performed consistently in this population. The final validated questionnaire included 6 items in the physical environment, 7 items in parent literacy habits, 5 items in child literacy habits, 4 items in parent-child interaction and 4 items in parental beliefs and perceived barriers towards early literacy domains. In general, the refined HLQ demonstrates adequate construct validity and may serve as a useful tool for examining home literacy influences on early literacy development.

### Parental responses to the questionnaire

Kerala has consistently been reported as the state with the highest literacy rate in India by National Statistical Office [NSO] (2020). In Kerala, 96.11 per cent of males and 92.07 per cent of females are literate, compared to 82.14 per cent of males and 65.46 per cent of females nationwide (Kerala State Planning Board, 2023). Reflecting this, all parents in the present study were literate and had at least a middle school education. The majority of the fathers were high school level graduates, and most mothers completed higher secondary education. Kerala is also recognized as a state with the highest levels of female education (Simister, 2011). The state’s generally high levels of literacy and education are reflected in the education profile of the current study sample.

The majority of the participants in the present study were from upper-lower and lower-middle socioeconomic backgrounds. According to the 2011 census, Kerala has relatively favorable socioeconomic and human welfare indices, with 27.93% of the total population being employed. In this current study also majority of the parents were employed, and less than 1% of fathers were unemployed.

#### Age and regularity of reading

In the present study, most parents reported introducing books to their children at the age of 3 years or later, which corresponds to previous Indian research. In particular, Aaruni et al. (2025) also observed that book introduction commonly occurs beyond 3 years, while Pandith et al. (2022) and Vrinda et al. (2022) reported a slightly wider range of 2–4 years. In contrast to Western reports (Honig and Shin, 2001; Price and Kalil, 2019; Kucirkova et al., 2012), the initiation of book exposure among Indian children appears to occur at a relatively later age. This may be due to the strong cultural emphasis on oral storytelling over early reading practices. For recent reading activity, nearly half of the respondents reported reading to their children on the previous day, consistent with earlier findings (Aaruni et al., 2025). Parents in this study typically engaged in reading activities with their children one to three times per week, with each session lasting for 10 min. It is comparable to reports by Pandith et al. (2022) and Kalia (2007). Still, it contrasts with findings from Aaruni et al. (2025), who documented considerably longer duration of reading sessions of at least half an hour in Malayalam-speaking older children aged 5 to 6 years. Higher reading frequencies are also documented in Western contexts (Bracken and Fischel, 2008). Zainiddinov and Habibov (2019) examined the typical amount of time mothers spend interacting with their children under 5 years old in a number of Central Asian nations, including time spent reading books, telling stories, practicing counting and recognizing items. Turkmenistan and Uzbekistan had the most mother-child interaction time, followed by Kazakhstan, Kyrgyzstan, and Tajikistan. Picture storybooks were identified as the most commonly preferred reading material, followed by Malayalam children’s magazines. Across the surveyed households, the availability of children’s books was generally limited, with most families owning fewer than ten. This level of access is considerably lower than what has been reported in several other cultural settings (Sénéchal et al., 1998; Stephenson et al., 2008). This highlights the substantial influence of sociocultural and environmental factors on the presence of children’s books within the home environment.

#### Physical environment

Within the physical environment domain, only a small proportion of parents reported using board books to teach basic concepts such as shapes and colors and even fewer indicated providing puzzle books. The majority of households possessed concept books on body parts, fruits, vegetables, numbers, and the alphabet. These materials were typically placed within children’s reach, and many homes had a designated space for storing books. The pattern observed here further reflects that Indian households tend to rely more on children’s magazines and short story books rather than on using puzzle books or board books, which are more commonly used in Western contexts. Similarly, Malo (2024) reported that print materials available in Assamese homes typically included textbooks, fiction, and newspapers, indicating considerable variation in the types of reading resources present across Indian families.

Research from economies classified as upper-middle-income typically reports high rates of home access to educational materials. For instance, according to Wickramasekara et al. (2014), over 90% of third graders in Sri Lanka reported they have newspapers and storybooks at home.

According to two studies, 78% of children in Lebanon and almost all children in Jordan had four or more different kinds of toys and reading materials (Queen Rania Foundation, 2017; Save the Children, 2017). On the other hand, research indicates that less than half of families in low to lower-middle-income countries like India, Nepal, and Indonesia possess print materials, storybooks, and number toys (Bhattacharjea et al., 2011; Bora et al., 2018; Mayasya, 2017).

#### Parent literacy habits

With regard to parental literacy habits, a large majority of parents reported having a personal interest in reading during leisure time. Nearly half of the respondents indicated that they use varied tones or expressive voices while reading. Many parents also mentioned that they trace words with their fingers or point to them as they read. Almost all parents shared that they enjoy discussing the stories or books they read with other family members, reflecting a home environment where reading is valued and integrated into daily interactions. Another interesting report by parents was that they like to reread books they have enjoyed previously, which may further reinforce a culture of reading within the household. In addition, the majority reported that their children frequently observe them reading, offering children consistent exposure to positive literacy behaviors. When children observe their parents engaging enthusiastically in the reading process, it fosters a positive attitude toward books and learning. Supportive and enjoyable reading interactions can enhance children’s interest in stories, thereby increasing their motivation to explore books independently (Nan and Tian, 2025).

#### Child literacy habits

Parents in this study report that their children showed a strong interest in reading-related activities, aligning with the findings of Kalia and Vagh (2008) and Pandith et al. (2022). Many parents also noted that their children frequently requested to be read to, which is consistent with the observations of Sénéchal et al. (1998) and Bracken and Fischel (2008), suggesting that children actively initiate joint storybook reading. Informants further described their children as curious and self-motivated, often asking questions when they did not understand a story. Parents also recognized that their children were able to follow along effectively during shared reading sessions.

#### Parent-child interaction for reading-related activities

The results indicate a mixed pattern of parent-child interactions, with certain practices widely adopted and others occurring less often. Nearly half of the respondents reported that they relate stories to real-life situations to enhance children’s understanding. The majority of the parents stated that they explain concepts to their children while watching television together. Parent conversation during shared screen time is associated with higher curiosity in children (Shah et al., 2021). Only a limited proportion of parents create new stories or songs for their children, suggesting that many prefer to rely on readily available materials rather than generating their own. Pandith et al. (2022) also reported in their study that certain practices, such as creating their own stories or encouraging children to make up their own, were not commonly observed among most Indian parents. Additionally, fewer than half of the parents only encourage their children to read signboards or product labels in everyday settings. This may be due to the perception that children are too young to recognize or understand environmental print, or that parents may not fully realize the importance of these early print awareness opportunities. Together, these findings highlight areas where parent-child literacy interactions could be further strengthened to support early literacy development.

#### Parental beliefs and perceived barriers toward early literacy

Findings from this study indicate that most parents believed that children should be introduced to reading at an early age and emphasized that parental involvement substantially contributes to children’s reading development and language proficiency. Theoretical perspectives consistently emphasize that joint literacy activities foster children’s language, reading, writing, and broader school readiness skills (Magen-Nagar and Firstater, 2019). The barriers reported were that their children were unwilling to sit for reading. Engaging toddlers and young children in reading activities can be challenging, as they often exhibit limited sustained attention or an apparent lack of interest. Others perceived that the absence of a calm and peaceful home literacy environment limited the opportunities for shared reading. Swain et al. (2017) reported that the limited physical space within the home hinders the creation of a quiet and supportive reading environment, thereby reducing opportunities for meaningful shared reading interactions. Additionally, time constraints emerged as a barrier, where parents find difficulty to engage with children for shared reading activities. Parental fatigue associated with prolonged workdays can significantly reduce both the regularity and quality of shared reading practices, despite parents’ intentions to engage (Swain et al., 2017). Time constraints arising from work, household duties, and extracurricular schedules often limit book-sharing opportunities. Consequently, opportunities for parent-child interaction may be reduced, affecting the consistency of literacy routines at home (Nan and Tian, 2025).

## Conclusion

Preliminary findings indicate that the tool is valid and comprehensive for evaluating home environmental factors. The questionnaire can be used by speech-language pathologists to better understand children’s literacy context. The study also shed light on parents’ reading practices, beliefs, and physical environment, emphasizing the importance of assessing these factors to support children’s early literacy development.

## Limitations

Response and recall bias may have occurred in the study because it relied on parent-reported responses. The reliability of the home literacy environment measures may have been impacted by parents’ inadvertent overreporting of positive literacy behaviors or underreporting of less frequent literacy encounters. Although home literacy experiences begin in early childhood, this study included responses of parents only from children aged 3 to 6 years. Future studies may investigate literacy experiences during earlier developmental periods to better understand their influence on subsequent early literacy development.

## Data Availability

The raw data supporting the conclusions of this article will be made available by the authors, without undue reservation.
