# Supplementary material for: Development and validation of a home literacy questionnaire for Malayalam-speaking preschoolers
Source: Front Psychol. 2026 Jun 15;17:1841280. doi: 10.3389/fpsyg.2026.1841280 (PMC13312784; doi:10.3389/fpsyg.2026.1841280)
Supplement: Supplementary file 1 [file Data_Sheet_1.pdf]

മാതാപിതാക്കൾക്കുള്ള ചോദ്യാവലി

ഈ ചോദ്യാവലി കുട്ടികളുടെ വീടുകളിലെ ആദ്യകാല സാക്ഷരത മനസ്സിലാക്കുന്നതിന് വേണ്ടിയാണ്. നിങ്ങൾ നൽകുന്ന വിവരങ്ങൾ ഗവേഷണ ആവശ്യത്തിന് മാത്രമേ ഉപയോഗിക്കുകയുള്ളൂ. ചോദ്യാവലി ശ്രദ്ധയോടെ വായിച്ച് എല്ലാ ചോദ്യങ്ങൾക്കും ഉചിതമായ ഉത്തരങ്ങൾ നൽകുവാൻ താല്പര്യപ്പെടുന്നു.

കുട്ടിയുടെ പേര്: പ്രായം/ലിംഗഭേദം:

കുട്ടിയുടെ ജനന തിയ്യതിയും വർഷവും:

മാതൃഭാഷ: തീയതി:

വീട്ടിൽ ഏതെല്ലാം ഭാഷകൾ ഉപയോഗിക്കുന്നു:

സ്കൂളിന്റെ പേര്:

കുട്ടിയുടെ ക്ലാസ് / സ്റ്റാൻഡേർഡ്:

വീടിന്റെ വിലാസം:

ബന്ധപ്പെടാനുള്ള നമ്പർ:

അച്ഛന്റെ പേര്:

അച്ഛന്റെ വിദ്യാഭ്യാസ യോഗ്യത:

അച്ഛന്റെ ജോലി:

അമ്മയുടെ പേര്:

അമ്മയുടെ വിദ്യാഭ്യാസ യോഗ്യത:

അമ്മയുടെ ജോലി:

മാസവരുമാനം (ശരിയായത് ✓ ചെയ്യുക):

i) 40,430 രൂപയോ അതിൽ കൂടുതലോ മാസ വരുമാനം ഉണ്ട്
ii) 20, 210 – 40, 429 രൂപ മാസ വരുമാനം ഉണ്ട്
iii) 15-160 – 20,209 രൂപ മാസ വരുമാനം ഉണ്ട്
iv) 10,110 – 15, 159 രൂപ മാസ വരുമാനം ഉണ്ട്
v) 6060 – 10, 109 രൂപ മാസ വരുമാനം ഉണ്ട്
vi) 2021 – 6059 രൂപ മാസ വരുമാനം ഉണ്ട്
vii) 2020 രൂപയോ അതിൽ കുറവോ മാസ വരുമാനം ഉണ്ട്

Vellithiruthi Thazhath Aaruni[1], Radhakrishnan Chella Perumal[2], Murugesan Krupa[3], Jayashree Chandrashekhar Shanbal[4], Arjunan Vasanthy Anjana[5]

Age & regularity of reading engagement

1. ഏത് പ്രായത്തിലാണ് നിങ്ങൾ നിങ്ങളുടെ കുട്ടിക്ക് പുസ്തകങ്ങൾ വായിച്ചു കൊടുക്കാൻ തുടങ്ങിയത്?
   a. ഒരു വയസ്സിനു താഴെ പ്രായം ഉള്ളപ്പോൾ
   b. 1-2 വയസ്സ്
   c. 2-3 വയസ്സ്
   d. 3 വയസ്സും അതിനു മുകളിലും

2. എപ്പോഴാണ് നിങ്ങൾ അവസാനമായി നിങ്ങളുടെ കുട്ടിക്ക് പുസ്തകങ്ങൾ വായിച്ചുകൊടുത്തത് ?
   a. ഇന്നലെ
   b. കുറച്ച് ദിവസം മുമ്പ്
   c. ഒരാഴ്ച മുമ്പ്
   d. ഒരു മാസം മുമ്പ്

3. ആഴ്ചയിൽ എത്ര ദിവസം നിങ്ങൾ നിങ്ങളുടെ കുട്ടിക്ക് പുസ്തകം ഉറക്കെ വായിച്ചു കൊടുക്കുന്നു?
   a. ആഴ്ചയിൽ 1-3 തവണ
   b. ആഴ്ചയിൽ 3-5 തവണ
   c. ആഴ്ചയിൽ 5-7 തവണ
   d. ഒരിക്കലുമില്ല

4. ഒരു ദിവസം എത്ര സമയം വരെ നിങ്ങൾ കുട്ടിക്ക് പുസ്തകം വായിച്ചു കൊടുക്കുന്നു?
   a. < 5 മിനിറ്റ്
   b. 10 മിനിറ്റ്
   c. അര മണിക്കൂർ
   d. ഒരു മണിക്കൂറിന് മീതെ

5. ഏത് തരം പുസ്തകമാണ് നിങ്ങളുടെ കുട്ടിക്ക് നിങ്ങൾ വായിച്ച് കൊടുക്കാൻ ഇഷ്ടപ്പെടുന്നത്?
   a. ചെറുകഥാ പുസ്തകങ്ങൾ
   b. ചിത്രകഥാ പുസ്തകങ്ങൾ
   c. നാടോടി കഥകൾ / ഗുണപാഠ കഥകൾ
   d. കുട്ടികളുടെ മാസികകൾ (ഉദാ. കളിക്കുടുക്ക, ബാലരമ, ബാലഭൂമി)

6. നിങ്ങളുടെ വീട്ടിലെ കുട്ടികളുടെ പുസ്തകങ്ങളുടെ എണ്ണം എത്രയാണ്?
   a. ഒന്നുമില്ല.
   b. < 10 പുസ്തകങ്ങൾ
   c. < 20 പുസ്തകങ്ങൾ
   d. >20 പുസ്തകങ്ങൾ

Vellithiruthi Thazhath Aaruni[1], Radhakrishnan Chella Perumal[2], Murugesan Krupa[3], Jayashree Chandrashekhar Shanbal[4], Arjunan Vasanthy Anjana[5]

Physical environment

ഇനി പറയുന്ന ചോദ്യങ്ങൾ കുട്ടികൾക്ക് വായിക്കാനും എഴുതാനും ലഭ്യമായ മെറ്റീരിയലുകൾ ഉൾപ്പെടെയുള്ള ഭൗതിക പരിതസ്ഥിതിയുമായി ബന്ധപ്പെട്ടിരിക്കുന്നു. നിങ്ങളുടെ ഉത്തരം ഉണ്ട് എന്നാണെങ്കിൽ '✓' ഇല്ല എന്നാണെങ്കിൽ 'X' നൽകുക

S.No	ഇനങ്ങൾ	ഉണ്ട്	ഇല്ല
1.	നിറങ്ങളും ആകൃതികളും വലുപ്പങ്ങളുമെല്ലാം പഠിപ്പിക്കുന്നതിനായുള്ള ബോർഡ് ബുക്കുകൾ വീട്ടിൽ ലഭ്യമാണോ?		
2.	നിങ്ങളുടെ വീട്ടിൽ പസ്സിൽ പുസ്തകങ്ങൾ ഉണ്ടോ?		
3.	മൃഗങ്ങളുടെ പേരുകൾ, പഴങ്ങൾ, ശരീരഭാഗങ്ങൾ മുതലായവ പഠിപ്പിക്കുന്ന പുസ്തകങ്ങൾ നിങ്ങളുടെ വീട്ടിൽ ലഭ്യമാണോ?		
4.	നിങ്ങളുടെ വീട്ടിൽ അക്ഷരമാല, അക്കങ്ങൾ എന്നിവ പഠിപ്പിക്കുന്നതിനു ആവശ്യമായ പുസ്തകങ്ങൾ ഉണ്ടോ?		
5.	പുസ്തകങ്ങൾ കുട്ടിക്ക് എളുപ്പത്തിൽ ലഭ്യമാണോ?		
6.	പുസ്തകങ്ങൾ സൂക്ഷിക്കാൻ നിങ്ങളുടെ വീട്ടിൽ ഒരു നിശ്ചിത സ്ഥലം ഉണ്ടോ?		

Vellithiruthi Thazhath Aaruni[1], Radhakrishnan Chella Perumal[2], Murugesan Krupa[3], Jayashree Chandrashekhar Shanbal[4], Arjunan Vasanthy Anjana[5]

Parent literacy habits

ഇനി പറയുന്ന ചോദ്യങ്ങൾ നിങ്ങളുടെ വായന, എഴുത്ത് എന്നി പ്രവർത്തനങ്ങളുമായി ബന്ധപ്പെട്ടതാണ്. നിങ്ങളുടെ ഉത്തരം ഉണ്ട് എന്നാണെങ്കിൽ '✓' ഇല്ല എന്നാണെങ്കിൽ 'X' നൽകുക

S.No	ഇനങ്ങൾ	ഉണ്ട്	ഇല്ല
1.	നിങ്ങൾക്ക് പുസ്തകങ്ങൾ, പത്രങ്ങൾ, അല്ലെങ്കിൽ മാസികകൾ വായിക്കുന്നതിൽ വ്യക്തിപരമായ താൽപ്പര്യമുണ്ടോ?		
2.	നിങ്ങൾ പുസ്തകങ്ങളോ കഥകളോ വായിക്കുമ്പോൾ ശബ്ദത്തിൽ മാറ്റങ്ങൾ വരുത്തി വായിക്കാറുണ്ടോ?		
3.	നിങ്ങൾ പുസ്തകങ്ങളോ കഥകളോ വായിക്കുമ്പോൾ വിരലുകൊണ്ട് പിന്തുടരുകയോ വാക്കുകളിലേക്ക് വിരലുകൾ ചൂണ്ടിക്കാണിക്കുകയോ ചെയ്യാറുണ്ടോ?		
4.	നിങ്ങൾ വീട്ടിലെ മറ്റ് ആളുകളുമായി നിങ്ങൾ വായിച്ച കഥകളോ പുസ്തകങ്ങളോ സംബന്ധിച്ച് ചർച്ച ചെയ്യാറുണ്ടോ?		
5.	നിങ്ങൾക്ക് ഇഷ്ടപ്പെട്ട പുസ്തകങ്ങളോ ലേഖനങ്ങളോ വീണ്ടും വായിക്കാറുണ്ടോ?		
6.	നിങ്ങൾക്ക് മറ്റുള്ളവർക്ക് വേണ്ടി പുസ്തകങ്ങൾ ഉറക്കെ വായിച്ച് കൊടുക്കാൻ ഇഷ്ടമാണോ?		
7.	നിങ്ങൾ ഒരു പുസ്തകമോ പത്രമോ വായിക്കുന്നത് നിങ്ങളുടെ കുട്ടി കാണാറുണ്ടോ?		

Child's own literacy habits

ഇനി പറയുന്ന ചോദ്യങ്ങൾ വായനയിലും എഴുത്തിലുമുള്ള കുട്ടിയുടെ താൽപ്പര്യവുമായി ബന്ധപ്പെട്ടതാണ്. നിങ്ങളുടെ ഉത്തരം ഉണ്ട് എന്നാണെങ്കിൽ '✓' ഇല്ല എന്നാണെങ്കിൽ 'X' നൽകുക

S.No	ഇനങ്ങൾ	ഉണ്ട്	ഇല്ല
1.	നിങ്ങളുടെ കുട്ടി പുസ് തകങ്ങൾ വായിക്കാൻ താത്പര്യം കാണിക്കാറുണ്ടോ?		
2.	പുസ്തകങ്ങൾ ഉറക്കെ വായിച്ച് തരുവാൻ നിങ്ങളുടെ കുട്ടി നിങ്ങളോട് അഭ്യർഥിക്കാറുണ്ടോ?		
3.	കഥകൾ വായിക്കുമ്പോൾ നിങ്ങളുടെ കുട്ടി ഇടപഴുകാറുണ്ടോ?		
4.	ഒരു കഥ വ്യക്തമല്ലാത്തപ്പോൾ നിങ്ങളുടെ കുട്ടി ചോദ്യങ്ങൾ ചോദിക്കാറുണ്ടോ?		
5.	നിങ്ങൾ കഥ വായിച്ചുകേൾപ്പിക്കുമ്പോൾ നിങ്ങളുടെ കുട്ടി നിങ്ങളോടൊപ്പം പിന്തുടരാൻ ശ്രമിക്കാറുണ്ടോ?		

Parent-child interaction for language and literacy activities

ഇനി പറയുന്ന ചോദ്യങ്ങൾ നിങ്ങളുടെ കുട്ടിയുമായി നിങ്ങൾ ഭാഷാപഠനത്തിൽ എങ്ങനെ ഇടപഴകുന്നു എന്നതിനെക്കുറിച്ചാണ് . നിങ്ങളുടെ ഉത്തരം ഉണ്ട് എന്നാണെങ്കിൽ '✓' ഇല്ല എന്നാണെങ്കിൽ 'X' നൽകുക

S.No	ഇനങ്ങൾ	ഉണ്ട്	ഇല്ല
1.	കഥ വായിക്കുമ്പോൾ അത് കൂടുതൽ യാധ്യാർഥമാവാൻ കുട്ടിയുടെ ജീവിതവുമായി നിങ്ങൾ ബന്ധപ്പെടുത്താറുണ്ടോ?		
2.	വാക്കുകൾ / ശബ്ദങ്ങൾ ഉപയോഗിച്ച് നിങ്ങൾ പുതിയ കവിതകൾ സൃഷ്ടിക്കാറുണ്ടോ?		
3.	കുട്ടി ടി വി കാണുമ്പോൾ , കാണുന്ന പരിപാടിയെ കുറിച്ച് വിശദീകരിക്കാറുണ്ടോ?		
4.	ഉൽപ്പന്ന ടാഗുകൾ, സൈൻ ബോർഡുകൾ എന്നിവ വായിക്കാൻ നിങ്ങൾ നിങ്ങളുടെ കുട്ടിയെ പ്രോത്സാഹിപ്പിക്കാറുണ്ടോ?		

Vellithiruthi Thazhath Aaruni[1], Radhakrishnan Chella Perumal[2], Murugesan Krupa[3], Jayashree Chandrashekhar Shanbal[4], Arjunan Vasanthy Anjana[5]

Parental beliefs and perceived barriers towards Literacy

ഇനി പറയുന്ന പ്രസ്താവനകൾ വായനയോടും എഴുത്തിനോടും ബന്ധപ്പെട്ട നിങ്ങളുടെ വിശ്വാസങ്ങളും നേരിടുന്ന വെല്ലുവിളികളും സംബന്ധിച്ചവയാണ്. നിങ്ങളുടെ ഉത്തരം ഉണ്ട് എന്നാണെങ്കിൽ '✓' ഇല്ല എന്നാണെങ്കിൽ 'X' നൽകുക

S.No	ഇനങ്ങൾ	ഉണ്ട്	ഇല്ല
1.	ഞാൻ എന്റെ കുട്ടിക്ക് കഥാപുസ്തകങ്ങൾ വായിച്ചു കൊടുക്കാറില്ല കാരണം അവൻ അല്ലെങ്കിൽ അവൾ ഒരിടത്ത് നിശ്ചലമായി ഇരിക്കില്ല. .		
2.	വീട്ടിൽ ശാന്തമായ സ്ഥലമില്ലാത്തതിനാൽ ഞാൻ എന്റെ കുട്ടിക്ക് പുസ്തകങ്ങൾ വായിച്ചു കൊടുക്കാറില്ല.		
3.	ഞാൻ എന്റെ കുട്ടിക്ക് പുസ്തകങ്ങൾ വായിച്ചു കൊടുക്കാറില്ല കാരണം അതിനായി സമയം കണ്ടെത്താൻ എനിക്ക് ബുദ്ധിമുട്ടാണ്.		
4.	എന്റെ കുട്ടി വളരെ ചെറുപ്പത്തിൽ തന്നെ പുസ്തകങ്ങൾ വായിക്കേണ്ടതില്ല എന്ന് ഞാൻ വിശ്വസിക്കുന്നു.		

Vellithiruthi Thazhath Aaruni[1], Radhakrishnan Chella Perumal[2], Murugesan Krupa[3], Jayashree Chandrashekhar Shanbal[4], Arjunan Vasanthy Anjana[5]
