# Supplementary material for: Development and validation of a home literacy questionnaire for Malayalam-speaking preschoolers
Source: Front Psychol. 2026 Jun 15;17:1841280. doi: 10.3389/fpsyg.2026.1841280 (PMC13312784; doi:10.3389/fpsyg.2026.1841280)
Supplement: Supplementary file 2 [file Table_1.pdf]

Supplementary File

Table 1 I-CVI and S-CVI/Ave for HLQ domain

Domain	Items	I-CVI	S-CVI/Ave
**Age and regularity of reading**	Item 1	1	0.93
	Item 2	0.6	
	Item 3	1	
	Item 4	1	
	Item 5	1	
	Item 6	1	
**Physical environment**	Item 1	1	0.8
	Item 2	0.6	
	Item 3	1	
	Item 4	1	
	Item 5	0.6	
	Item 6	0.6	
**Parent literacy habits**	Item 1	1	1
	Item 2	1	
	Item 3	1	
	Item 4	1	
	Item 5	1	
	Item 6	1	
	Item 7	1	
	Item 8	1	

**Child literacy habits**	Item 1	1	1
	Item 2	1	
	Item 3	1	
	Item 4	1	
	Item 5	1	
	Item 6	1	
	Item 7	1	
	Item 8	1	
**Parent-child interaction for literacy-related activities**	Item 1	1	0.95
	Item 2	1	
	Item 3	1	
	Item 4	1	
	Item 5	0.6	
	Item 6	1	
	Item 7	1	
	Item 8	1	
**Parental beliefs & perceived barriers towards early literacy**	Item 1	1	0.9
	Item 2	1	
	Item 3	0.6	
	Item 4	1	
	Item 5	0.6	
	Item 6	1	

Item 7	1
Item 8	1
